# In Vitro Sonodynamic Therapeutic Effect of Polyion Complex Micelles Incorporating Titanium Dioxide Nanoparticles

**DOI:** 10.3390/nano7090268

**Published:** 2017-09-11

**Authors:** Satoshi Yamamoto, Masafumi Ono, Eiji Yuba, Atsushi Harada

**Affiliations:** Department of Applied Chemistry, Graduate School of Engineering, Osaka Prefecture University, 1-1 Gakuen-cho, Naka-ku, Sakai, Osaka 599-8531, Japan; st108078@edu.osakafu-u.ac.jp (S.Y.); Masafumi_Ono@hisamitsu.co.jp (M.O.); yuba@chem.osakafu-u.ac.jp (E.Y.)

**Keywords:** Titanium dioxide nanoparticles, sonodynamic therapy, polyion complex micelles

## Abstract

Titanium dioxide nanoparticles (TiO_2_ NPs) can act as sonosensitizers, generating reactive oxygen species under ultrasound irradiation, for use in sonodynamic therapy. For TiO_2_ NPs delivery, we prepared polyion complex micelles incorporating TiO_2_ NPs (TiO_2_ NPs-PIC micelles) by mixing TiO_2_ NPs with polyallylamine bearing poly(ethylene glycol) grafts. In this study, the effects of polymer composition and ultrasound irradiation conditions on the sonodynamic therapeutic effect toward HeLa cells were evaluated experimentally using cell viability evaluation, intracellular distribution observation, and a cell staining assay. TiO_2_ NPs-PIC micelles with widely distributed features induced a significant decrease in cell viability under ultrasound irradiation. Furthermore, prolonging the irradiation time killed cells more effectively than did increasing the ultrasound power. The combination of TiO_2_ NP-PIC micelles and ultrasound irradiation was confirmed to induce apoptotic cell death.

## 1. Introduction

Titanium dioxide (TiO_2_) can act as a photosensitizer and is known to generate reactive oxygen species (ROS), including OH and HO_2_ radicals, superoxide anions (O^2−^), hydrogen peroxide (H_2_O_2_), and ^1^O_2_, under ultraviolet (UV) irradiation (less than 390 nm) [1,2,3,4]. UV-irradiated TiO_2_ nanoparticles (NPs) have shown a cell-killing effect toward HeLa cells [5]. However, the clinical use of TiO_2_ NPs is hampered because UV light cannot deeply penetrate human tissue, and TiO_2_ NPs have poor dispersion stability at physiological pH [6,7]. Shimizu et al. found that TiO_2_ generates ROS under ultrasound irradiation (39 kHz) [8], although the ultrasound frequency (39 kHz) was too low for clinical applications. Additionally, sonicating TiO_2_ NPs at a clinically appropriate frequency that allows deep body invasion (1 MHz) also showed an effective decrease in cell viability and inhibited tumor growth in vivo when TiO_2_ NP suspension was directly injected into tumor [9]. This indicated the availability of TiO_2_ NPs in sonodynamic therapy (SDT) and showed that developing a carrier system that could deliver TiO_2_ NPs into cells by improving their dispersion stability under physiological conditions was required for effective SDT.

We have focused on the charge properties of surface OH groups on TiO_2_ NPs. The isoelectric point of TiO_2_ NPs with an anatase crystal structure is 6.2, meaning that TiO_2_ NPs are negatively charged at neutral pH [10]. Polyion complex (PIC) micelles incorporating TiO_2_ NPs (TiO_2_ NP-PIC micelles) were successfully prepared using polyallylamine bearing poly(ethylene glycol) grafts (PAA-g-PEG) [11], in which the micelles were formed through electrostatic interaction as a driving force, and van der Waals force and hydrophobic interaction were also stabilized as a result of polyion complex formation. Although bare TiO_2_ NPs have poor solubility against water at physiological pH, the incorporation of TiO_2_ NPs into the micelles provided a remarkable improvement in dispersion stability. It was confirmed that ultrasound irradiation to HeLa cells treated by TiO_2_ NP-PIC micelles induced a decrease in cell viability through ^1^O_2_ generation. The cell viability decreased as irradiation time increased. When other irradiation conditions were kept constant, the decrease in cell viability was dependent on irradiation time. Furthermore, this decrease in cell viability was completely inhibited by the presence of glutathione, which is a radical scavenger, demonstrating that the cell-killing effect was due to ROS generated by ultrasound irradiation of the TiO_2_ NP-PIC micelles. In this study, we evaluated the effects of polymer composition and sonication time on the cell-killing effect of the TiO_2_ NP-PIC micelles. Furthermore, it was confirmed that the cell-killing effect of the TiO_2_ NP-PIC micelles was induced by apoptotic cell death.

## 2. Results and Discussion

TiO_2_ NP-PIC micelles were prepared using four types of PAA-g-PEG bearing PEGs of different molecular weights (Mn = 2000 and 5000) and grafting densities (13 and 26 mol % for PEG2000, and 12 and 21 mol % for PEG5000), named 2k13, 2k26, 5k12, and 5k21, respectively. The mean diameter, polydispersity index, zeta potential, and composition (polymer/TiO_2_ *w*/*w*) were determined, as summarized in Table 1. For the mean diameter and zeta-potential, it was difficult to compare with bare TiO_2_ NPs due to their poor dispersion stability at physiological pH. The compositions were controlled by the molecular weight (Mn) of the PEG grafts, with PEG grafts of Mn 2000 and 5000 giving polymer/TiO_2_ *w*/*w* ratios of 2 and 4, respectively. The mean diameter tended to grow large, such that the PEG graft Mn was also large and the PEG graft content was high. Importantly, all prepared TiO_2_ NP-PIC micelles had almost neutral zeta potentials, suggesting that electrically neutral PEG grafts surrounded the micellar surface.

**Figure 1:**
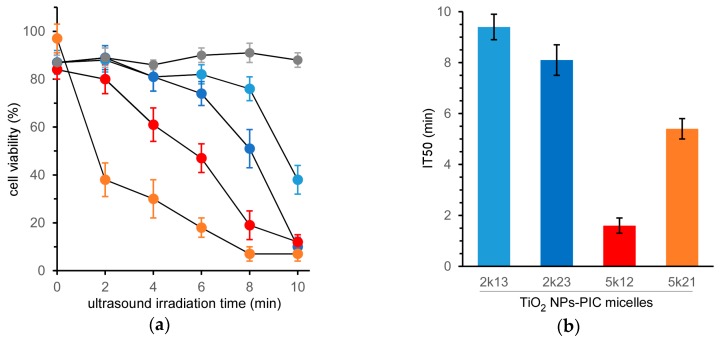
Cell-killing effect of TiO_2_ NP-PIC micelles under ultrasound irradiation. (**a**) Effect of ultrasound irradiation time on viability of HeLa cells treated with TiO_2_ NP-PIC micelles. (**b**) Half maximal inhibitory time of ultrasound irradiation (IT50) of TiO_2_ NP-PIC micelles. 2k13, 2k26, 5k12, and 5k21 micelles and without the micelles are represented by light blue, blue, orange, red, and gray symbols, respectively. Ultrasound irradiation was performed for varying times (frequency: 1.0 MHz; power: 0.5 W/cm^2^; duty cycle: 10%).

The cell-killing effect of the TiO_2_ NP-PIC micelles toward HeLa cells was evaluated by MTT (3-(4,5-di-methylthiazol-2-yl)-2,5-diphenyltetrazolium bromide) assay at various ultrasound irradiation times (Figure 1a). Micelles without ultrasound irradiation (ultrasound irradiation time = 0) maintained high cell viability and demonstrated negligible cytotoxicity. Prolonging ultrasound irradiation induced a decrease in cell viability, showing an obvious difference in the cell-killing effect among the micelles. Additionally, ultrasound irradiation to the cells without the treatment of the TiO_2_ NP-PIC micelles did not induce the decrease in cell viability as shown in Figure 1a, although ultrasound irradiation to a solvent without TiO_2_ NPs induce the generation of solvent radicals, i.e., H and OH radicals that can combine to give hydrogen and hydrogen peroxide in the case of water [12,13]. This suggests that the main cytotoxic species in ROS generated by ultrasound irradiation might be singlet oxygen. The half maximal inhibitory time of ultrasound irradiation (IT50) values, as an indication of the cell-killing effect under ultrasound irradiation, were determined from Figure 1a for each micelle, as shown in Figure 1b. There was a 5.7-fold difference between the IT50 values of the most effective 5k12 micelles and the least effective 2k13 micelles.

**Figure 2:**
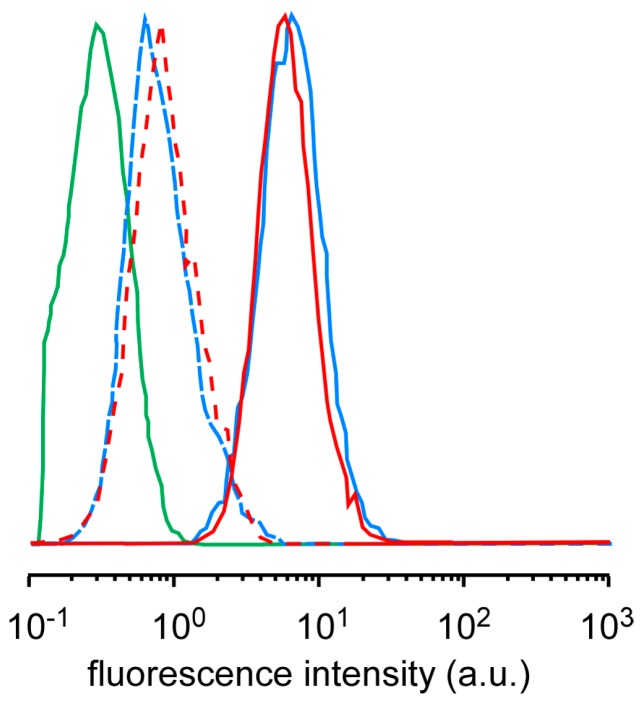
Flow cytometry analysis of ^1^O_2_ generation of TiO_2_ NP-PIC micelles (red line, 5k12 micelles; light blue line, 2k13 micelles) with (solid line) and without (dashed line) ultrasound irradiation. (frequency: 1.0 MHz; power: 1.0 W/cm^2^; irradiation time: 2 min; duty cycle: 10%).

**Figure 3:**
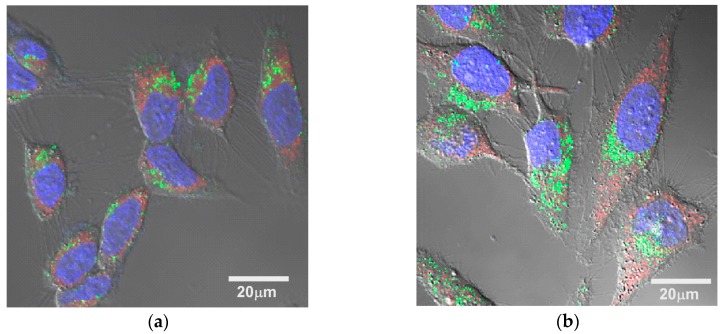
Confocal laser scanning microscopy images overlaid with differential interference contrast images of HeLa cells treated with (**a**) 2k13 and (**b**) 5k12 micelles for 24 h of incubation. Micelles were prepared using FITC-labeled TiO_2_ NPs. Nuclei and mitochondria were stained with Hoechst and MitoTracker Red, respectively.

The difference in the cell-killing effect among the micelles might be due to the difference in ROS generation, cellular uptake, and intracellular distribution. Singlet oxygen sensor green (SOSG) has been used as a probe to confirm that the ultrasound irradiation of TiO_2_ NP-PIC micelles increased the amount of ^1^O_2_ generation in proportion to the irradiation time and the ultrasound power, and that there was no difference among the micelles [11]. The amounts of ^1^O_2_ generation for 5k12 micelles and 2k13 micelles were compared using SOSG by flow cytometry (Figure 2). For both micelles, the fluorescence intensity of HeLa cells treated by the mixture of micelles and SOSG were slightly increased even without ultrasound irradiation, suggesting that the comparable amount of SOSG were taken up into the cells. The ultrasound irradiation to HeLa cells treated with the mixture of micelles and SOSG provided significant increase in fluorescence intensity, indicating that TiO_2_ NP-PIC micelles could generate ^1^O_2_ in the cells, and there was no difference in the fluorescence intensity between 5k12 micelles and 2k13 micelles. Furthermore, the cellular uptake of micelles was already evaluated by flow cytometry [11]. The TiO_2_ NPs uptake increased in an incubation time-dependent manner, suggesting that cellular uptake occurred via an endocytosis pathway, with no meaningful difference in TiO_2_ NPs uptake among the micelles. Consequently, TiO_2_ NP-PIC micelles could generate ^1^O_2_ in the cells even after 24 hours of incubation with HeLa cells, and the generated amount of ^1^O_2_, which is the main cytotoxic species among ROS, might be comparable among micelles. Therefore, to explain the difference in the cell-killing effect among the micelles, the intracellular distribution of the micelles, especially their distribution to mitochondria, was compared between the most effective 5k12 micelles and least effective 2k13 micelles using fluorescein 5-isothiocyanate (FITC)-labeled TiO_2_ NPs. ROS damage to mitochondria is known to effectively induce cellular apoptosis [14]. The intracellular distributions of the micelles were compared by laser scanning microscopy (Figure 3), in which the mitochondria were stained and observed. Mitochondria, identified by red fluorescence, were widely distributed in the cytoplasm. For both micelles, green fluorescence dots were observed in the cytoplasm, with most green fluorescence overlapping with red fluorescence. However, it should be noted that 5k12 micelles were more widely distributed in the cytoplasm than the 2k13 micelles despite the comparable amount of TiO_2_ NP-PIC micelle uptake into the cells. This meant that 5k12 micelles were likely to cause ROS damage to more mitochondria. The difference in the cell-killing effect among the micelles shown in Figure 1 might be due to the difference in intracellular distribution of the TiO_2_ NP-PIC micelles.

**Figure 4:**
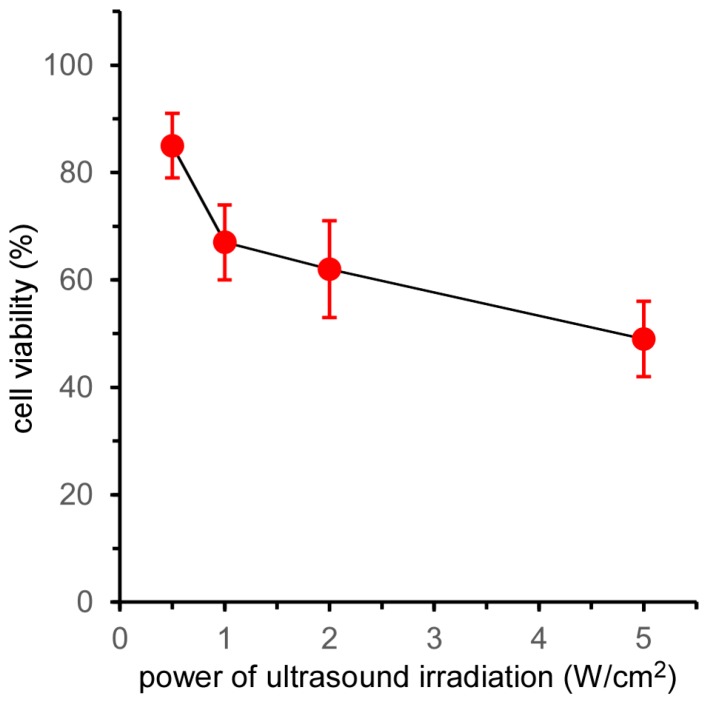
Effect of ultrasound irradiation power on the viability of HeLa cells treated by 5k21 micelles. Ultrasound irradiation was performed using varying ultrasound power (frequency: 1.0 MHz; irradiation time: 2 min; duty cycle: 10%).

The effect of ultrasound power on the cell-killing effect of 5k21 micelles was evaluated by MTT assay (Figure 4). The cell viability at a power of 0.5 W/cm^2^ after 2 min of irradiation was the same as that shown in Figure 1a. Increasing the ultrasound power resulted in a decrease in cell viability. However, the effect of ultrasound power was weak compared with that of irradiation time, with half the cells remaining alive at an ultrasound power of 5.0 W/cm^2^. As described above, the amount of ^1^O_2_ generated by ultrasound irradiation to TiO_2_ NP-PIC micelles increased in proportion with both the irradiation time and the ultrasound power [11]. By increasing the ultrasound power from 0.5 to 5.0 W/cm^2^, the generated amount of ^1^O_2_ increased 10-fold, but the cell viability decreased by approx. 50%. Furthermore, the cell viability in Figure 4 stopped falling at approx. 50%, with little decrease in cell viability observed when further increasing the ultrasound power. In contrast, prolonging the irradiation time from 2 to 10 min increased the amount of ^1^O_2_ generated five-fold and effectively decreased the cell viability to approx. 10%. These results indicated that prolonging the ultrasound irradiation time was more effective than increasing the ultrasound power to increase the sonodynamic therapeutic effect of TiO_2_ NP-PIC micelles. The diffusion of the micelles in the cytoplasm might participate in this difference between the effects of irradiation time and power. Due to high reactivity, the lifetime and diffusion distance of ROS in the cytoplasm are 10–40 ns and 10–20 nm, respectively [15], and these values were determined for ROS generated by photo-irradiation to photosensitizer. The reactivity of ROS is the same as those generated by ultrasound irradiation to TiO_2_ NPs, and ROS generated by ultrasound irradiation to TiO_2_ NP-PIC micelles inside the cells have comparable lifetime and diffusion distance of ROS generated by photo-irradiation to photosensitizer. Therefore, TiO_2_ NP-PIC micelles can only damage the limited mitochondria existing near them during ultrasound irradiation. Accordingly, prolonging the irradiation time, which increases the diffusion area, might result in an increased cell-killing effect, while the increase in the ultrasound power might not be effective. This agreed with the results shown in Figure 1 and Figure 3, in which micelles distributed widely in the cytoplasm exhibited effective cell-killing.

**Figure 5:**
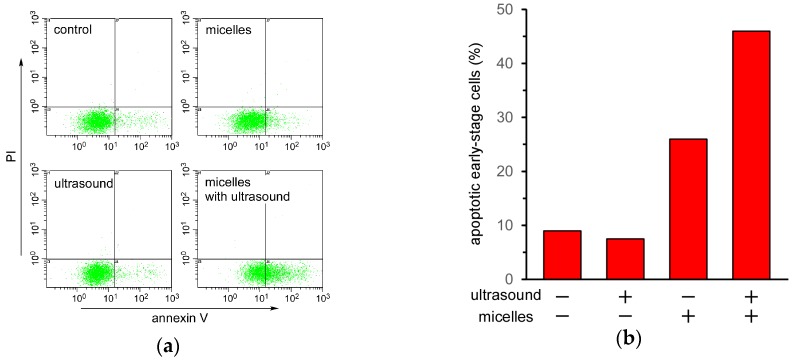
Evaluation of cell death mechanism for HeLa cells treated with 5k21 micelles with and without ultrasound irradiation. (**a**) Flow cytometry of HeLa cells doubly stained with annexin V-FITC and PI. (**b**) Percentages of apoptotic early-stage cells under various treatments. HeLa cells were incubated with the micelles for 24 h, after which ultrasound irradiation (frequency: 1.0 MHz; irradiation time: 6 min; power: 0.5 W/cm^2^; duty cycle: 10%) was performed.

Finally, the mechanism of cell death induced by ultrasound irradiation of the TiO_2_ NP-PIC micelles was evaluated by an annexin V and propidium iodide (PI) double staining assay using flow cytometry. In the case of apoptotic cells, their phospholipid membrane asymmetry is lost, leading to the exposure of phosphatidylserine (PS) at the cellular surface, a process that can be monitored using annexin V. Annexin V can identify apoptotic cells with the exposed PS, since annexin V is a Ca^2+^-dependent phospholipid-binding protein with a high affinity for PS. The stage of apoptosis can be distinguished using both FITC-labeled annexin V and PI. At the late stage of apoptosis, the permeability of the plasma membrane increases, and PI can bind to cellular DNA by moving across the cell membrane. Therefore, late-stage cells are stained with both PI and annexin V, whereas early-stage cells are stained with only annexin V. Figure 5a shows the flow cytometry of HeLa cells under various treatments. The cell count in the lower right region, in which the cells were stained with only annexin V, increased when treated with the micelles and further increased under ultrasound irradiation. This increase in cell count in the lower right region under ultrasound irradiation indicated that ultrasound irradiation induced apoptosis in HeLa cells treated with TiO_2_ NP-PIC micelles. Cell death induced by a combination of TiO_2_ NPs and ultrasound irradiation has been reported to be due to apoptosis. Yamaguchi et al. reported that water-dispersed TiO_2_-PEG induced apoptotic cell death in human glioblastoma cell line U251 under ultrasound irradiation [16]. Furthermore, Ninomiya et al. reported that TiO_2_ NPs modified with pre-S1/S2 proteins, which are part of the L protein from the hepatitis B virus with a high affinity toward hepatocyte, induced apoptotic cell death in human hepatoma HepG2 cells under ultrasound irradiation [17]. Therefore, it is fitting that cell death induced by TiO_2_ NP-PIC micelles under ultrasound irradiation was due to apoptosis, not necrosis.

## 3. Materials and Methods 

### 3.1. Materials

Four kinds of PAA-g-PEG with the same PAA main chain (DP = 160), bearing PEGs of different molecular weights (Mn = 2000 and 5000) and grafting densities (13 and 26 mol % for PEG2000, and 12 and 21 mol % for PEG5000), were synthesized according to our previous report [18]. These graft copolymers were abbreviated as 2k13, 2k26, 5k12, and 5k21, respectively. TiO_2_ NP dispersion with an anatase crystal structure (10 nm, pH < 3) was purchased from Ishihara Sangyo Kaisha, Ltd. (Osaka, Japan). Fluorescein 5-isothiocyanate (FITC)-labeled TIO_2_ NPs were prepared according to our previous report [11]. MitoTracker Red CMXRos and Hoechst 33342 were purchased from Thermo Fisher Scientific Inc. (Waltham, MA, USA). An Annexin-V-FLUOS staining kit was purchased from Roche Diagnostics GmbH (Mannheim, Germany). Fetal calf serum (FCS) was purchased from Biowest (Riverside, MO, USA). Dulbecco’s modified Eagle’s medium (DMEM) was purchased from Nissui Pharmaceutical (Tokyo, Japan). Singlet oxygen sensor green (SOSG) was purchased from Invitrogen (Eugene, OR, USA). All reagents were used without further purification.

### 3.2. Preparation of TiO_2_ NP–PIC Micelles

TiO_2_ NP dispersion and PAA-g-PEG aqueous solutions were mixed with the weight ratio of polymer to TiO_2_ (polymer/TiO_2_) fixed at 10. The mixing solutions (pH < 3) were neutralized using aqueous NaOH. Free polymer was removed by ultrafiltration using a USY-20 ultrafiltration unit (molecular weight cut off: 200,000; Toyo Roshi, Ltd. (Tokyo, Japan), and the solvent was also exchanged with phosphate-buffered saline (PBS). The final micelle composition, i.e., the weight ratio of polymer and TiO_2_, was determined using thermogravimetric/differential thermal analysis.

### 3.3. Physicochemical Characterization of TiO_2_ NP-PIC Micelles

DLS and laser-Doppler electrophoresis measurements were carried out at 25 °C using an ELS-8000 (Otsuka Electronics Co., Ltd., Osaka, Japan) instrument equipped with an He/Ne ion laser (*λ* = 633 nm). In DLS measurements, and the mean diameter was calculated using the Stokes-Einstein equation [19]. Laser-Doppler electrophoresis (Hercules, CA, USA) was employed as a technique to measure particle velocity. The electrophoretic mobility was determined from frequency shifts, which is the difference between scattered light and original beam, caused by the Doppler effect. The zeta potential was calculated using the Smoluchowski equation [19]. TG/DTA measurements were carried out using a TG8120 instrument (Rigaku, Tokyo, Japan). The samples were measured under an N_2_ atmosphere from room temperature to 550 °C at a heating rate of 10 °C/min and calibrated using Al_2_O_3_ as a standard sample.

### 3.4. Experiments Using Cultured Cells

HeLa cells were seeded in 100 μL of DMEM supplemented with 10% FCS in each well of a 96-well plate at 1 × 10^4^ cells for 1 day. Micelle solutions were gently added to the cells and incubated at 37 °C for 24 h. In the case of the confirmation of ^1^O_2_ generation, the mixture of micelle solutions including SOSG were gently added to the cells. The cells were washed with PBS and 100 μL of DMEM supplemented with 10% FCS. Ultrasound irradiation was performed using an ultrasound probe (*Φ* 6 mm) with a size similar than that of a well in the 96-well plate. The probe was immersed into culture media, and the distance between probe and the bottom of 96-well plate was fixed to 7 mm. After sonication, 6 μL of MTT (3-(4,5-dimethylthiazol-2-yl)-2,5-diphenyl tetrazolium bromide) solution was added to each well, and the plates were incubated 37 °C for 3 h, followed by the addition of 100 μL of 2-isopropanol containing 0.1 M HCl. The number of viable cells was determined by absorbance at 570 nm. For the annexin V and propidium iodide (PI) double staining assay, the cells were incubated for 6 h after ultrasound irradiation and then stained using an Annexin-V-FLUOS staining kit (Mannheim, Germany). After staining, the cells were detached from the surface of the dish using trypsin, and the cellular fluorescence was evaluated by flow cytometry (EPICS XL, Beckman Coulter, Inc. Brea, CA, USA).

## 4. Conclusions

TiO_2_ NP-PIC micelles exhibited a cell-killing effect toward HeLa cells through ^1^O_2_ generation under ultrasound irradiation. The wide intracellular distribution of TiO_2_ NP-PIC micelles and prolonged ultrasound irradiation time provided effective cell-killing corresponding to the wide distribution of mitochondria in the cytoplasm, suggesting that TiO_2_ NP-PIC micelles might induce apoptosis through singlet oxygen generation by ultrasound irradiation. It is expected that TiO_2_ NP-PIC micelles might become a clinically available sonodynamic therapy system via intravenous injection through the combination with high intensity focused ultrasound irradiation.

## Figures and Tables

**Table 1:** Characterization of TiO_2_ NP-PIC micelles prepared using various kinds of poly(ethylene glycol) grafts (PAA-g-PEG).

PAA-g-PEG	Mean Diameter (nm) ^1^	Zeta Potential (mV) ^2^	Composition (Polymer/TiO_2_ *w*/*w*) ^3^
2k13	61	−0.1	2.1
2k26	86	1.1	1.8
5k12	89	2.9	4.0
5k21	132	1.6	4.6

^1^ Values determined using dynamic light scattering (DLS). ^2^ Values determined using laser-Doppler electrophoresis. ^3^ Values calculated using thermogravimetric/differential thermal analysis (TG/DTA).
